# A systematic review and meta-analysis of the bacterial profile of ready-to-eat vegetable salads across Africa

**DOI:** 10.3389/fmicb.2026.1802185

**Published:** 2026-06-03

**Authors:** Kawooya Abubaker, Jesca Nakavuma, Chinyere Nkemjika Anyanwu, Emmanuel Eilu, Hussein Mukasa Kafeero, Julius Tibyangye, Danladi Makeri, Saheed Adekunle Akinola

**Affiliations:** 1 Department of Microbiology and Immunology, Kampala International University Western Campus, Bushenyi, Uganda; 2 College of Veterinary Medicine, Animal Resources and Biosecurity, Makerere University, Kampala, Uganda; 3 Institute of Allied Health Sciences, Clarke International University, Kampala, Uganda; 4 Department of Medical Microbiology, Faculty of Health Sciences, Habib Medical School, Islamic University in Uganda, Mbale, Uganda; 5 Department of Microbiology and Parasitology, School of Medicine and Pharmacy, College of Medicine and Health Sciences, University of Rwanda, Butare, Rwanda

**Keywords:** food safety, foodborne pathogens, microbiological quality of vegetables, ready-to-eat foods, vegetable salads

## Abstract

**Background:**

Ready-to-eat vegetable salads are increasingly consumed across Africa due to their convenience and perceived health benefits. However, these foods are often prepared without terminal heat treatment, making them potential vehicles for foodborne pathogens. Evidence of the burden and distribution of bacterial contamination in ready-to-eat vegetable salads across Africa remains fragmented. Therefore, this study aimed to perform a systematic review and meta-analysis of the prevalence of bacterial pathogens isolated from ready-to-eat vegetable salads across Africa.

**Methods:**

We searched PubMed, Scopus, and Google Scholar on 25 September 2025. Guided by the Preferred Reporting Items for Systematic Reviews and Meta-Analyses (PRISMA 2020) guidelines, eligible African studies reporting bacterial isolation from ready-to-eat vegetable salads were analyzed using random-effects models. Heterogeneity, publication bias, and single study influence on pooled prevalence were assessed. While this review was not prospectively registered in PROSPERO, we searched the registry to confirm no similar reviews existed and adhered strictly to PRISMA reporting standards.

**Results:**

Thirty-one (*n* = 31) studies were included. Gram-negative bacteria predominated among reported isolates. Meta-analysis was restricted to bacterial species reported in at least five studies to ensure stability of pooled estimates. *Escherichia coli* (40%; 95% CI: 20–64%) and *Salmonella* spp. (32%; 95% CI: 16–54%) were the most frequently reported organisms. Among Gram-positive bacteria, *Bacillus cereus* (28%; 95% CI: 14–48%) and *Staphylococcus* spp. (26%; 95% CI: 13–47%) were commonly identified.

**Conclusion:**

Ready-to-eat vegetable salads across Africa are frequently contaminated with bacterial communities, including both pathogenic and non-pathogenic organisms. This finding highlights the need for strengthened food safety practices and routine microbiological surveillance.

## Introduction

Ready-to-eat (RTE) vegetable salads are increasingly consumed worldwide due to their nutritional value, freshness, and convenience (Łepecka et al., 2022; Mir et al., 2018). They form a key component of healthy diets recommended for the prevention of non-communicable diseases (World Health Organization, 2023; Hoy et al., 2019). However, their minimal processing and the absence of terminal heat treatment make them particularly vulnerable to microbial contamination (Albu et al., 2024; Zernadji et al., 2025). The World Health Organization estimates that unsafe food causes approximately 600 million illnesses and 420 000 deaths annually, with the highest burden occurring in low- and middle-income countries (World Health Organization, 2021). Fresh produce and RTE foods are major contributors to this burden because pathogens introduced at any stage of the supply chain, production, irrigation, handling, or retail can persist until consumption (Bhatia et al., 2024; Alegbeleye et al., 2018). As global demand for minimally processed foods continues to rise, microbial contamination of RTE vegetable salads has emerged as an important food safety concern (Balali et al., 2020; Bhatia et al., 2024; Abubaker et al., 2026).

Several bacterial pathogens are of particular significance in RTE vegetable salads. *Escherichia coli*, including Shiga-toxin–producing strains, serves both as an indicator of fecal contamination and as a direct cause of diarrheal disease and haemolytic-uraemic syndrome (Luna-Guevara et al., 2019; Yussuf et al., 2025). *Salmonella* spp. is among the most common causes of foodborne outbreaks globally and has been associated with raw produce in many regions (Popa and Papa, 2021; Mkangara, 2023). *Listeria monocytogenes* poses a severe risk to pregnant women, neonates, and immunocompromised individuals because of its ability to proliferate under refrigeration (EFSA Panel on Biological Hazards et al., 2018; Yangchen et al., 2025). *Staphylococcus aureus* can produce enterotoxins responsible for staphylococcal food poisoning (Grispoldi et al., 2021; Cieza et al., 2024; Sosah and Donkor, 2025), while other opportunistic and environmental bacteria, such as *Klebsiella, Enterobacter*, and *Pseudomonas* species, are also frequently detected, often exhibiting multidrug resistance (Mahami et al., 2023; Asfaw et al., 2023). The presence of these organisms in salads reflects contamination from irrigation water, soil, handlers, or equipment and has significant implications for public health, given their potential to cause sporadic illness and outbreaks as well as to transmit antimicrobial-resistant strains through the food chain (Alegbeleye et al., 2018; Viana et al., 2025; Balali et al., 2020).

In Africa, changing dietary habits, rapid urbanization, and the proliferation of informal food markets have driven increased consumption of RTE vegetable salads in street settings, restaurants, and institutional catering (Casari et al., 2022). Studies across Nigeria (Makinde et al., 2023; Adesakin et al., 2020), Ghana (Ahiabor et al., 2024; Anthonia et al., 2025), Kenya (Koech et al., 2024; Hoffmann et al., 2023), Tanzania (Kayombo and Mayo, 2018; Yussuf et al., 2025; Magambo et al., 2024), and South Africa (Christison et al., 2008; Richter et al., 2022) report high bacterial loads and frequent detection of pathogenic species, often exceeding international microbiological limits. Nonetheless, available data remain fragmented and difficult to generalize. A previous continental meta-analysis of foodborne pathogens in foods from selected African countries by Paudyal et al. (2017) conducted almost a decade ago provided valuable baseline information but requires an update. Moreso only a small proportion of studies on vegetables and fruits were included. This limited representation of African countries underscores a substantial evidence gap regarding the microbial safety of RTE vegetable salads, a product category that is uniquely exposed to contamination because it is consumed raw, has high water activity, and depends heavily on post-harvest hygiene.

The absence of a focused, continent-wide synthesis on the bacterial profile of RTE vegetable salads constrains risk assessment and policy formulation. This systematic review and meta-analysis therefore consolidates the fragmented evidence, quantifying the pooled prevalence of key bacterial pathogens and documenting bacterial diversity associated with contamination.

## Methods

### Search strategy and inclusion criteria

A comprehensive literature search was conducted on 25 September 2025 to identify studies reporting bacterial contamination of ready-to-eat (RTE) vegetable salads in Africa. The search strategy followed the Preferred Reporting Items for Systematic Reviews and Meta-Analyses (PRISMA 2020) guidelines. We searched two databases, PubMed and Scopus; the search engine Google Scholar; as well as reference lists of relevant articles and reviews. Google Scholar was searched via Harzing's Publish or Perish (Windows GIU Edition) 8.19.5300.9483, retrieving the first 1000 most relevant records due to platform limitations. Search terms were developed using free-text keywords related to RTE vegetable salads and bacterial contamination and adapted for each database and applied independently to ensure comprehensive coverage. The Boolean operators AND and OR were used to combine terms within and between concepts. The final search string applied is presented in Supplementary material 1. Both peer-reviewed journal articles and gray literature were considered. The titles and abstracts of all retrieved records were screened, and potentially eligible full texts were reviewed against the inclusion criteria.

### Studies were included if they

- Reported primary isolation of bacteria from RTE vegetable salads (defined as raw or minimally processed vegetable mixtures consumed without further cooking)
- Were conducted within any African country
- Provided quantitative data on bacterial prevalence or count

### Studies were excluded if they

- Investigated cooked or heat-treated foods, non-vegetable-based RTE foods
- Lacked microbiological data
- Reported non-bacterial contamination
- Were reviews, commentaries, or lacked extractable data relevant to bacterial contamination

Although this review was not prospectively registered or supported by a standalone published protocol, we searched the proposed topic keywords in Prospero to ensure we were not duplicating the study. The conduct of the study was guided by the PRISMA 2020 statement. All stages of the review, including study selection, data extraction, and analysis, were performed in accordance with the PRISMA guideline to ensure methodological consistency and reduce the risk of bias.

### Study selection

**Figure 1:**
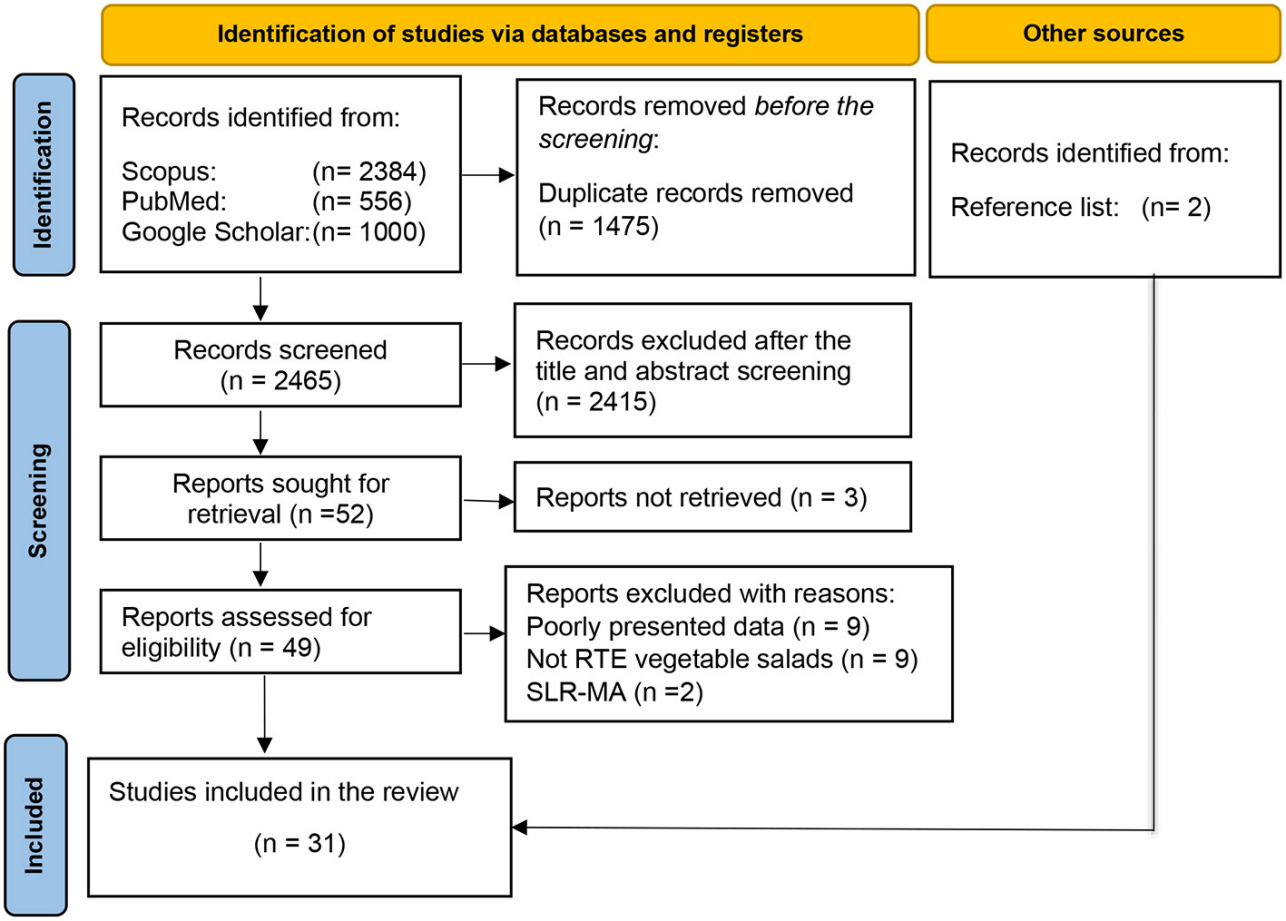
Preferred reporting Items for Systematic Literature Review and Meta-Analysis (PRISMA) Study Selection Flowchart (Page et al., 2021).

Records retrieved from the PubMed and Scopus databases were exported as CSV files and imported into Mendeley Desktop for deduplication. Title and abstract screening and full-text assessment were conducted independently in duplicate by two reviewers (KA and DM) for relevance against the inclusion criteria. Studies reporting bacterial isolation or detection from RTE vegetable salads in African countries were considered potentially eligible. Full texts of all potentially relevant articles were then assessed independently by the same reviewers. Discrepancies at either stage were documented in Microsoft Excel logs and through discussion and, where necessary, consultation with a third reviewer. A PRISMA 2020 flow diagram (Figure 1) was used to document the screening process, including the number of records identified, screened, excluded, and included.

### Data extraction

A standardized data extraction form was developed in Microsoft Excel and piloted on a subset of included studies to ensure consistency. The following information was extracted from each study: author(s), year of publication, country of study, sample type, bacterial species isolated, methods of isolation, and bacterial counts (prevalence data defined as presence/absence). Data extraction was performed independently by two reviewers, EE and SAA, and cross-checked by all authors for accuracy, and disagreements were resolved by consensus.

### Risk of bias assessment

**Figure 2:**
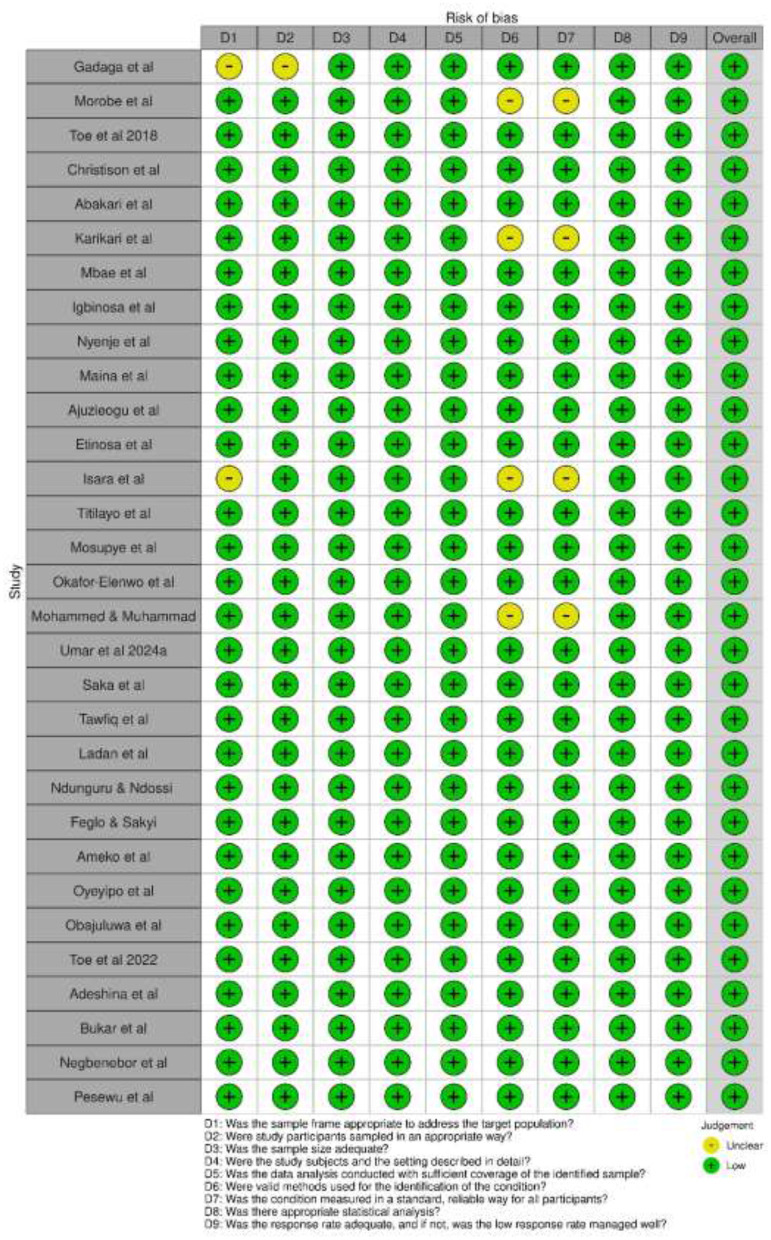
Risk of bias assessment.

Risk of bias and methodological quality of the included studies were assessed using the Joanna Briggs Institute (JBI) Critical Appraisal Checklist for Studies Reporting Prevalence and visualized using the ROBVIS tool (Figure 2). The checklist evaluates nine methodological items, including the appropriateness of the sampling frame, sampling method, sample size adequacy, description of study setting and participants, coverage of data analysis, validity of bacterial identification methods, appropriateness of statistical analysis, identification of relevant subpopulations, and management of response rates. Each item was rated as Yes, No, Unclear, or Not Applicable in accordance with JBI guidance. For overall risk-of-bias judgment, study-level ratings were derived by considering the collective performance across all checklist items. Studies were categorized as having low, moderate, or high risk of bias based on the extent to which methodological limitations were likely to influence prevalence estimates. Assessments were conducted independently by two reviewers (KA and DM), with discrepancies resolved through discussion and consensus.

### Statistical analysis

Descriptive analysis of study characteristics, including country, study setting, sample type, and bacterial species detected, was performed using Microsoft Excel (2019). The pooled prevalence (presence/absence) of bacterial contamination in ready-to-eat vegetable salads was estimated using the random-effects model in the R statistical environment to account for between-study variability. To stabilize variance and appropriately handle proportions close to 0 or 1, prevalence data were transformed using the Freeman-Tukey double arcsine transformation prior to pooling. This approach is widely recommended for meta-analysis of proportions to reduce the influence of extreme values and improve the robustness of pooled estimates. Heterogeneity across studies was assessed using the I^2^ statistic with 95% confidence intervals (CI) and interpreted as follows: low ( ≤ 25%), moderate (26–75%), and high (≥76%) (Christison et al., 2008). To evaluate potential publication bias, funnel plot asymmetry was visually inspected and complemented by Egger's regression test. A leave-one-out sensitivity analysis was conducted to assess the influence of individual studies on the pooled prevalence estimates. Bacterial diversity and distribution were analyzed descriptively, with results presented as frequencies and percentages. All statistical analyses were performed using R version 4.5.1.

## Results

### Study selection

A total of 3,940 bibliographies were retrieved cumulatively from Scopus and PubMed databases and the Google Scholar search engine. Of these, 1,475 duplicates were removed, and the remaining 2,465 underwent title and abstract screening to determine eligibility. Of the 2,465, we screened 52 eligible studies, of which 3 (*n* = 03) could not be retrieved as full text, leaving us with 49 articles, which we further screened for all components of the inclusion criteria. After the screening process, 29 studies met the inclusion criteria and were included in the review. To ensure comprehensive coverage, we performed a backward reference search of the eligible studies and identified 2 additional eligible records, resulting in a total number of included studies of 31. Study identification, screening, and inclusion were conducted following the PRISMA framework (Figure 1).

### Study characteristics

A total of 31 cross-sectional studies conducted in several African countries were included in this systematic review and meta-analysis (Table 1). Nigeria contributed the largest proportion of studies (51.61%; 16/31), followed by Ghana (16.13%; 5/31). Côte d'Ivoire, Kenya, and South Africa each accounted for 2 (6.45%) of the studies, while Botswana, Tanzania, and Zimbabwe were each represented by 1 study (3.23%). Regarding publication period, the earliest study on this topic across the continent was published in 1999. Subsequently, 8 studies (25.81%) were published between 2006 and 2012, 7 (22.58%) between 2013 and 2019, and 15 (48.39%) between 2020 and 2025.

**Table 1:** Study characteristics.

Authors	Year	Type of salad	Source	Method	Country	SS	TBI
Gadaga et al.	2008	Vegetable salads	Market	Culture	Zimbabwe	292	213
Morobe et al.	2009	Cabbage + Coleslow	Market	Culture	Botswana	500	47
Toe et al.	2018	Veg salads	Market	Culture	Côte d'Ivoire	306	436
Christison et al.	2008	Salads	Market	Culture	South Africa	35	5
Abakari et al.	2018	Salads	Market	Culture	Ghana	30	102
Karikari et al.	2022	salads	Market	Culture	Ghana	42	42
Mbae et al.	2018	Salads	Market	Culture	Kenya	39	20
Igbinosa et al.	2020	African Salads	Market	Culture	Nigeria	30	15
Nyenje et al.	2012	Veg salads	Market	Culture	S. Africa	42	33
Maina et al.	2021	Vegetables	Market	Culture	Kenya	91	175
Ajuzieogu et al.	2022	African Salads	Market	Culture	Nigeria	10	38
Etinosa et al.	2021	African Salads	Market	Culture	Nigeria	300	74
Isara et al.	2010	African Salads	Market	Culture	Nigeria	168	28
Titilayo et al.	2016	RTE Veg	Market	Culture	Nigeria	555	244
Mosupye et al.	1999	Salads	Market	Culture	S. Africa	12	3
Okafor-Elenwo et al.	2020	Salads	Market	Culture	Nigeria	3,840	3,840
Mohammed and Muhammad	2021	Vegetable salads	Random hawkers	Culture	Nigeria	200	133
Umar et al.	2024	Vegetable salads	Hawkers	Culture	Nigeria	100	36
Saka et al.	2022	Vegetable salads	Market	Culture	Nigeria	40	85
Tawfiq et al.	2024	Vegetable salads	Market	Culture	Nigeria	100	10
Ladan et al.	2025	Vegetable salads	Hawkers	Culture	Nigeria	240	36
Ndunguru and Ndossi	2020	Vegetable salads	Hawkers	Culture	Tanzania	14	15
Feglo and Sakyi	2012	Vegetable salads	Market	Culture	Ghana	10	17
Ameko et al.	2015	Vegetable salads	Hawkers	Culture	Ghana	150	235
Oyeyipo et al.	2022	Vegetable salads	Random canteens	Culture	Nigeria	15	51
Obajuluwa et al.	2020	Cabbage	Market	Culture	Nigeria	40	41
Toe et al.	2022	Vegetable salads	Markets	Molecular	Côte d'Ivoire	552	29
Adeshina et al.	2012	Vegetable salads	Restaurants	Culture	Nigeria	25	12
Bukar et al.	2010	Vegetable salads	Hawkers and food stalls	Culture	Nigeria	20	31
Negbenebor et al.	2019	Vegetable salads	Market	Culture	Nigeria	50	49
Pesewu et al.	2014	Mixed vegetable salad	Street vendors	Culture	Ghana	75	75

The bacterial profile identified across the included studies comprised both Gram-negative and Gram-positive bacteria (Table 2). Gram-negative organisms predominated, with *Proteus* spp. recording the highest total number of isolates (*n* = 2,284) and reported in 4 studies, followed by *Escherichia coli* (*n* = 688; 17 studies) and *Salmonella* spp. (*n* = 412; 18 studies). Other frequently detected Gram-negative bacteria included *Shigella* spp. (*n* = 175; 5 studies), *Klebsiella* spp. (*n* = 111; 11 studies), *Enterobacter* spp. (*n* = 66; 8 studies), *Citrobacter freundii* (*n* = 134; 6 studies), Pseudomonas aeruginosa (*n* = 44; 6 studies), Vibrio spp. (*n* = 99; 3 studies), *Aeromonas hydrophila* (*n* = 12; 2 studies), *Serratia marcescens* (*n* = 50; 2 studies), *Campylobacter* spp. (*n* = 2; 1 study), and *Campylobacter jejuni* (*n* = 36; 1 study). Among Gram-positive bacteria, *Bacillus cereus* was the most frequently isolated species, with a total of 1,412 isolates reported across 9 studies, followed by *Staphylococcus* spp. (*n* = 344; 10 studies) and Listeria spp. (*n* = 301; 4 studies).

**Table 2:** Bacterial profile of ready-to-eat vegetable salads.

Bacteria	TNI	NS	Studies
*Salmonella* spp.	412	18	(Gadaga et al., 2008; Christison et al., 2008; Abakari et al., 2018; Karikari et al., 2022; Igbinosa et al., 2024; Beshiru et al., 2020; Ajuzieogu et al., 2022; Isara et al., 2010; Mohammed and Muhammad, 2021; Umar et al., 2024; Maina et al., 2021; Saka et al., 2022; Shuraihu et al., 2025; Ndunguru and Ndossi, 2020; Ameko, 2012; Obajuluwa et al., 2021; Evelyne et al., 2022; Adeshina et al., 2012; Bukar et al., 2010)
*E. coli*	688	17	(Abakari et al., 2018; Karikari et al., 2022; Ajuzieogu et al., 2022; Maina et al., 2021; Saka et al., 2022; Shuraihu et al., 2025; Ndunguru and Ndossi, 2020; Obajuluwa et al., 2021; Adeshina et al., 2012; Bukar et al., 2010; Toe et al., 2018; Mbae et al., 2018; Igbinosa et al., 2020; Feglo and Sakyi, 2012; Oyeyipo et al., 2022; Helen Ehim et al., 2018; Pesewu et al., 2014)
*Klebsiella spp*.	111	11	(Ajuzieogu et al., 2022; Maina et al., 2021; Saka et al., 2022; Ndunguru and Ndossi, 2020; Obajuluwa et al., 2021; Igbinosa et al., 2020; Feglo and Sakyi, 2012; Oyeyipo et al., 2022; Helen Ehim et al., 2018; Pesewu et al., 2014; Nyenje et al., 2012)
*Staphylococcus* spp.	344	10	(Ajuzieogu et al., 2022; Isara et al., 2010; Saka et al., 2022; Ndunguru and Ndossi, 2020; Obajuluwa et al., 2021; Adeshina et al., 2012; Feglo and Sakyi, 2012; Helen Ehim et al., 2018; Pesewu et al., 2014; Okafor-Elenwo and Imade, 2020)
*B. cereus*	1,412	9	(Gadaga et al., 2008; Abakari et al., 2018; Isara et al., 2010; Saka et al., 2022; Ndunguru and Ndossi, 2020; Feglo and Sakyi, 2012; Okafor-Elenwo and Imade, 2020; Mosupye and von Holy, 1999; Pesewu et al., 2014)
*Enterobacter* spp.	66	8	(Ajuzieogu et al., 2022; Maina et al., 2021; Saka et al., 2022; Igbinosa et al., 2020; Oyeyipo et al., 2022; Helen Ehim et al., 2018; Nyenje et al., 2012)
*P. aeruginosa*	44	6	(Isara et al., 2010; Saka et al., 2022; Ameko, 2012; Adeshina et al., 2012; Igbinosa et al., 2020; Oyeyipo et al., 2022)
*C. freundii*	134	6	(Igbinosa et al., 2024; Maina et al., 2021; Saka et al., 2022; Feglo and Sakyi, 2012; Okafor-Elenwo and Imade, 2020; Helen Ehim et al., 2018)
*Shigella* spp.	175	5	(Abakari et al., 2018; Isara et al., 2010; Umar et al., 2024; Saka et al., 2022; Ameko, 2012)
*Listeria* spp.	301	4	(Christison et al., 2008; Nyenje et al., 2012; Morobe et al., 2009; Adesetan et al., 2013)
*Proteus* spp.	2284	4	(Maina et al., 2021; Nyenje et al., 2012; Okafor-Elenwo and Imade, 2020; Oyeyipo et al., 2022)
*Vibrio* spp.	99	3	(Ajuzieogu et al., 2022; Igbinosa et al., 2021; Bukar et al., 2010)
*A. hydrophila*	12	2	(Nyenje et al., 2012; Feglo and Sakyi, 2012)
*S. marcescens*	50	2	(Okafor-Elenwo and Imade, 2020; Obajuluwa et al., 2021)
*Campylobacter* spp.	2	1	(Mbae et al., 2022)
*C. jujeni*	36	1	(Mohammed and Muhammad, 2021)

TNI, Total Number of Isolates; NS, Number of studies.

### Pooled prevalences of bacteria isolated from RTEVS across Africa

We meta-analyzed pooled prevalences for bacterial isolates reported in at least five independent studies, including *Salmonella* spp., *E.coli*, Klebsiella spp., *Staphylococcus* spp., *B. cereus, Enterobacter* spp., *P. aeruginosa, C. freundii*, and *Shigella* spp. Isolates reported in fewer than five studies, including *Listeria* spp., *Proteus* spp., *Vibrio* spp., *A. hydrophila, S. marcescens*, and *Campylobacter* spp., were excluded from meta-analysis due to insufficient data and the risk of unstable pooled estimates.

**Figure 3:**
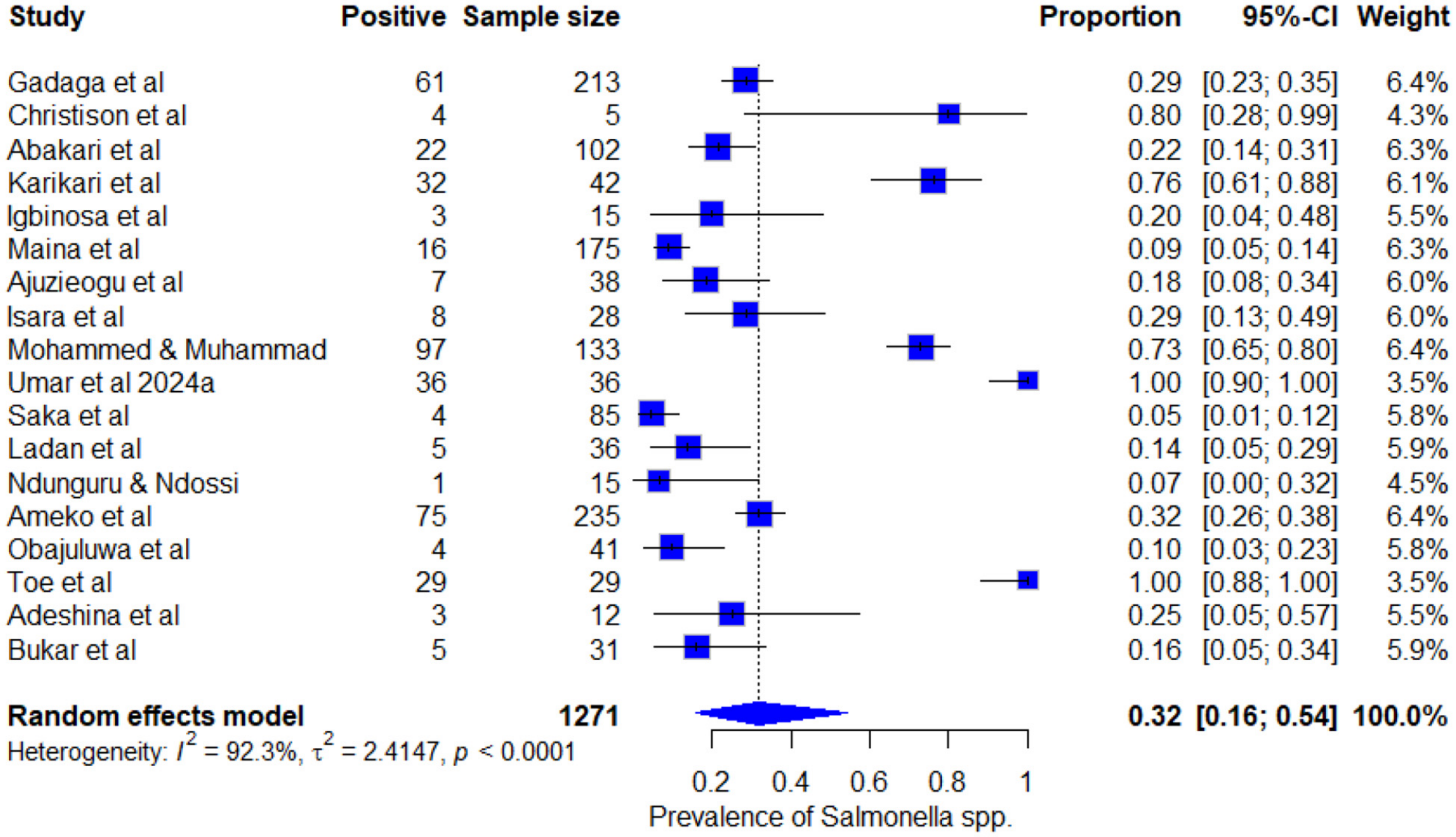
Pooled prevalence of *Salmonella* spp. isolated from ready-to-eat (RTE) vegetable salads across Africa. CI, confidence interval.

**Figure 4:**
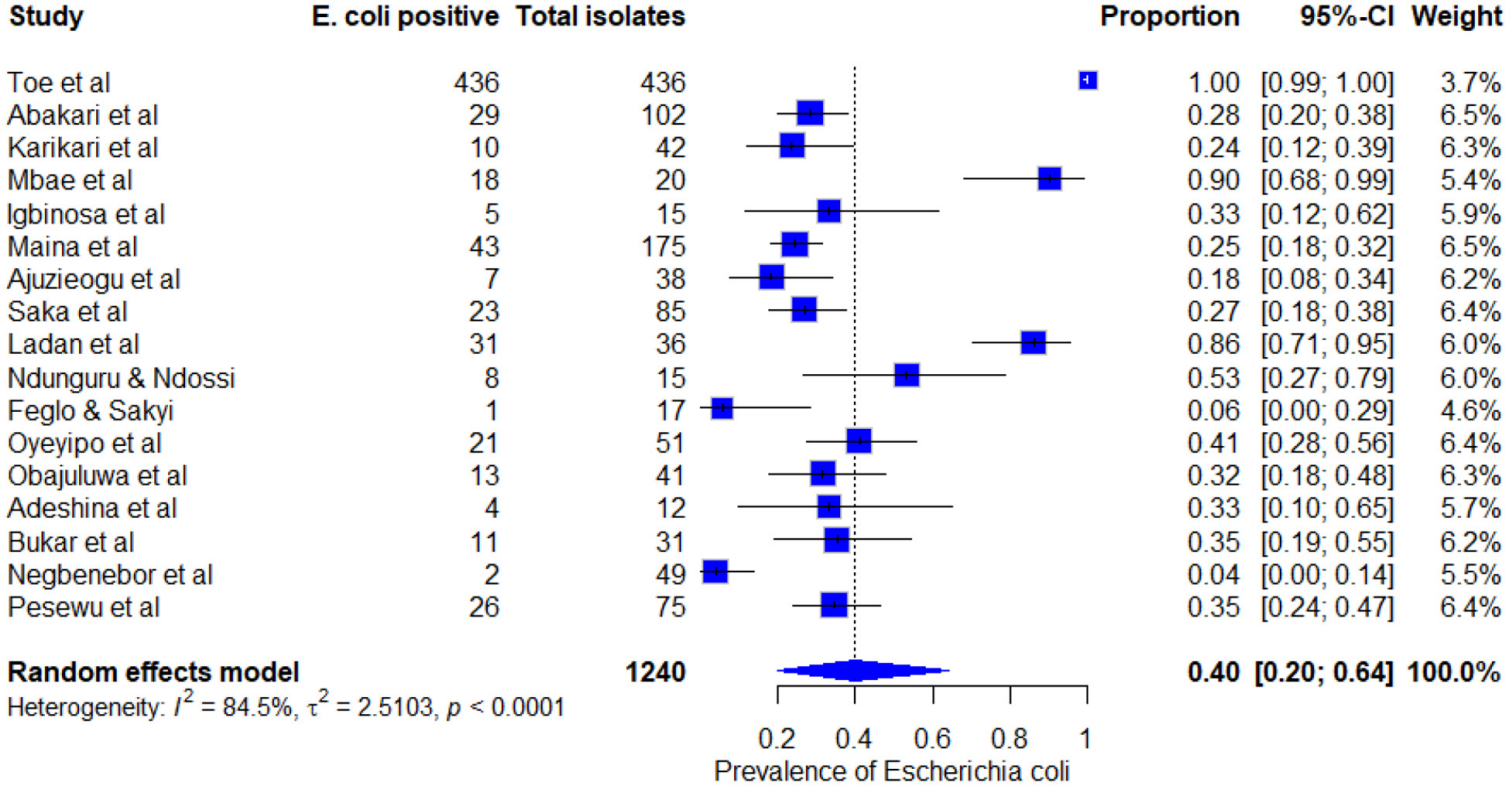
Pooled prevalence of *E. coli* isolated from ready-to-eat (RTE) vegetable salads across Africa. CI, confidence interval.

**Figure 5:**
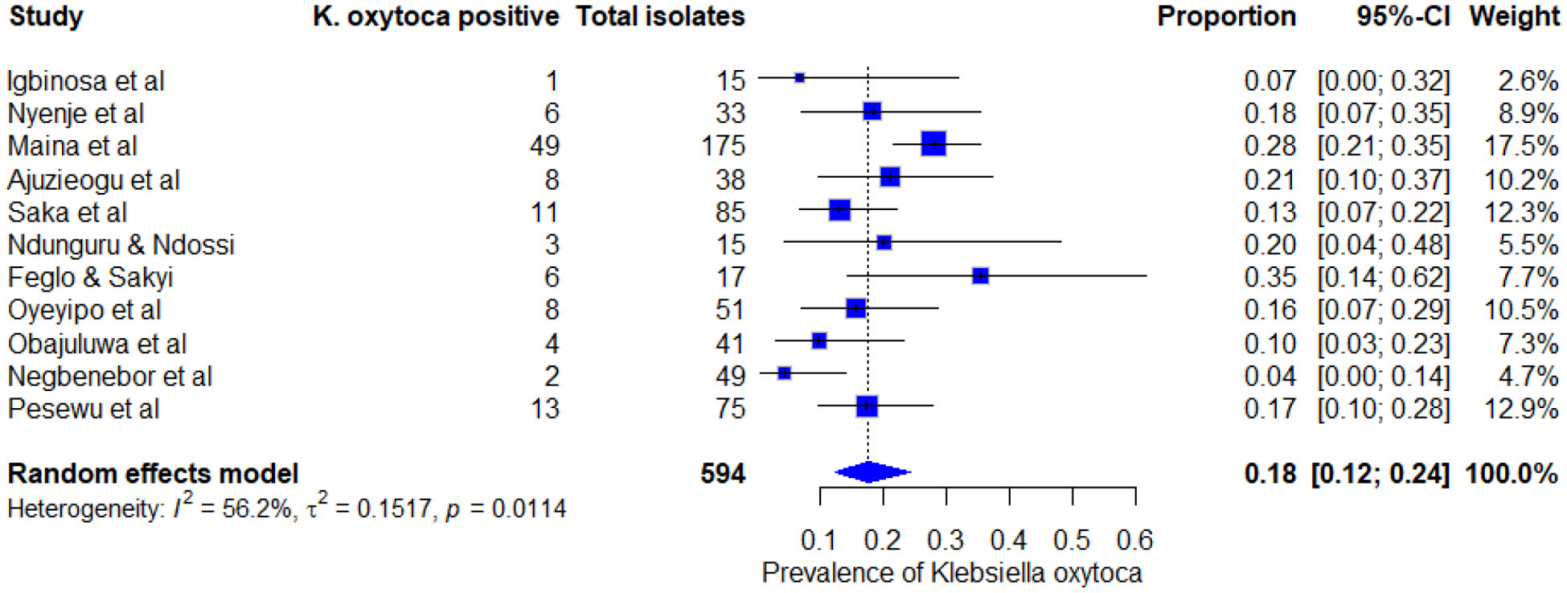
Pooled prevalence of K. oxytoca isolated from ready-to-eat (RTE) vegetable salads across Africa. CI, confidence interval.

**Figure 6:**
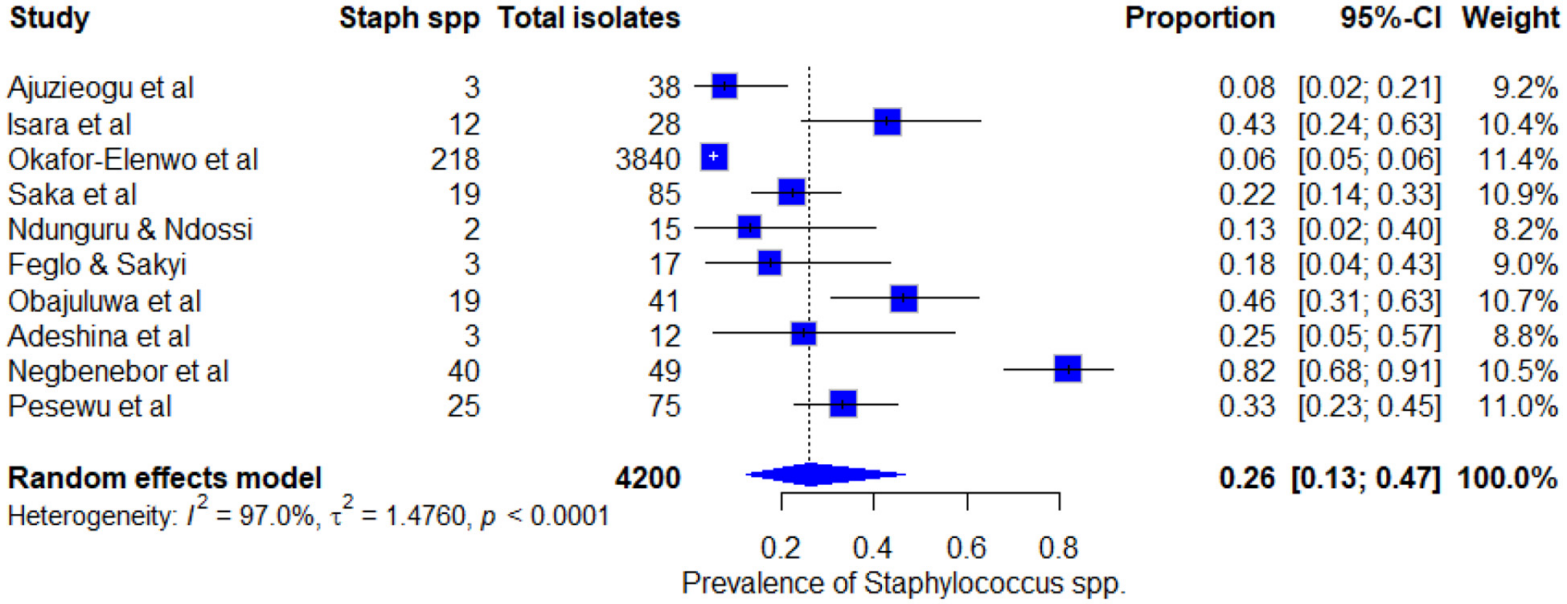
Pooled prevalence of *Staphylococcus* spp. isolated from ready-to-eat (RTE) vegetable salads across Africa. CI, confidence interval.

**Figure 7:**
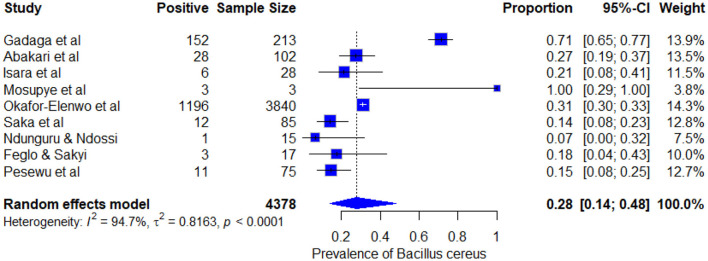
Pooled prevalence of *Bacillus cereus* isolated from ready-to-eat (RTE) vegetable salads across Africa. CI, confidence interval.

**Figure 8:**
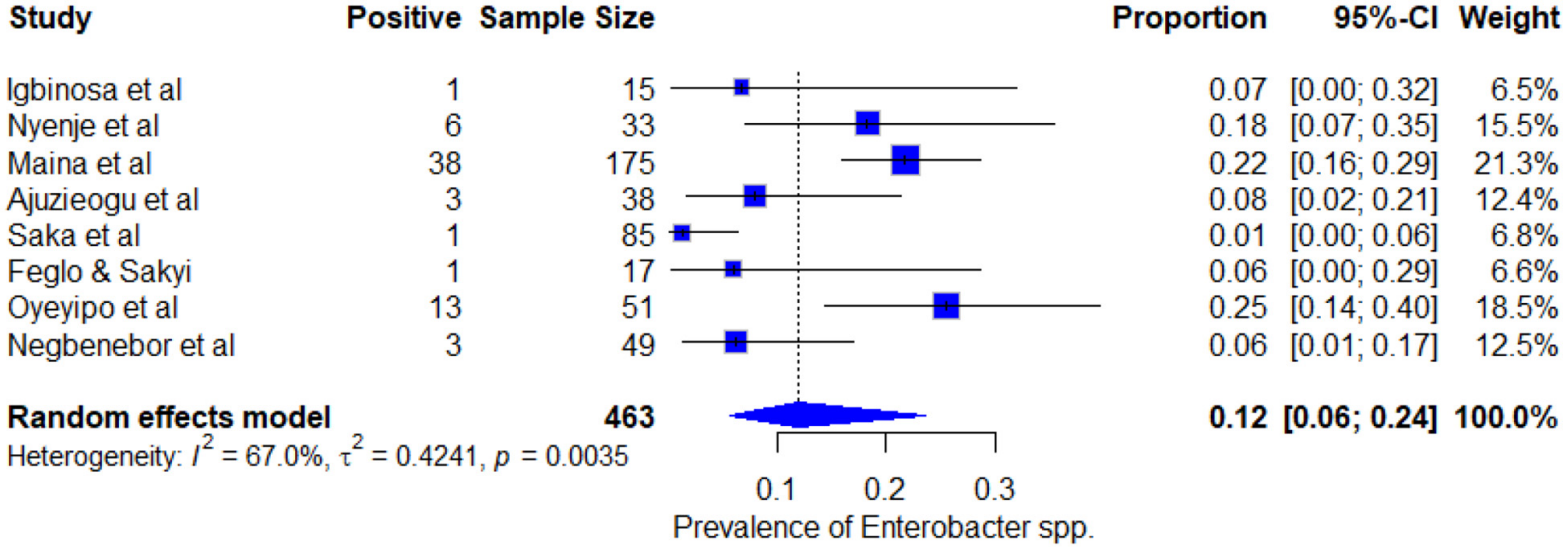
Pooled prevalence of *Enterobacter* spp. isolated from ready-to-eat (RTE) vegetable salads across Africa. CI, confidence interval.

**Figure 9:**
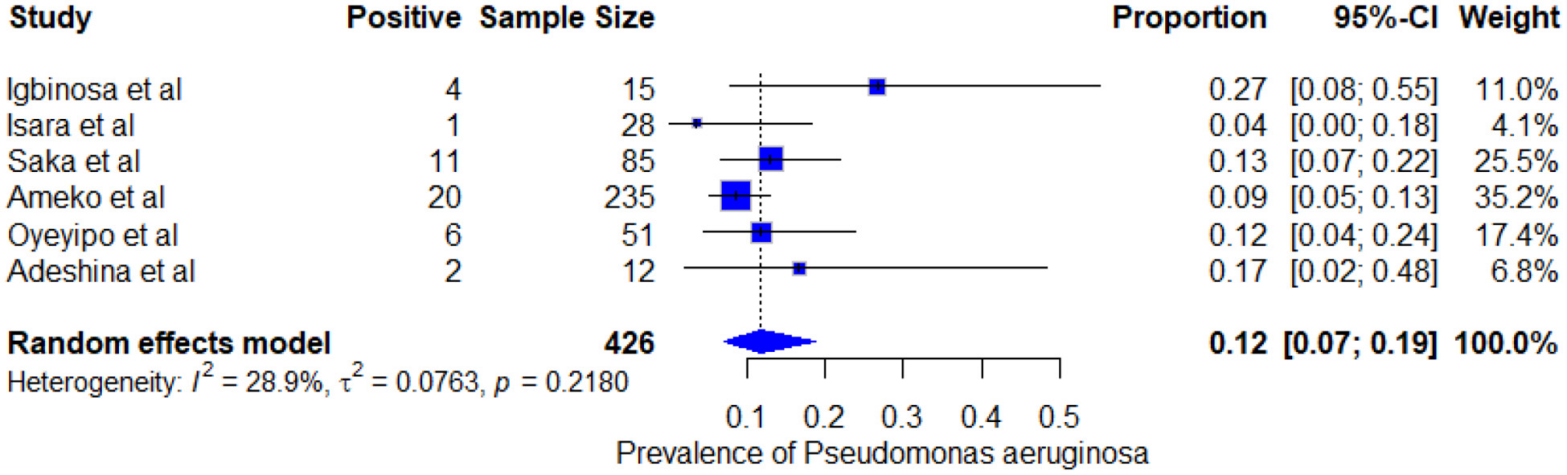
Pooled prevalence of *Pseudomonas aeruginosa* isolated from ready-to-eat (RTE) vegetable salads across Africa. CI, confidence interval.

**Figure 10:**
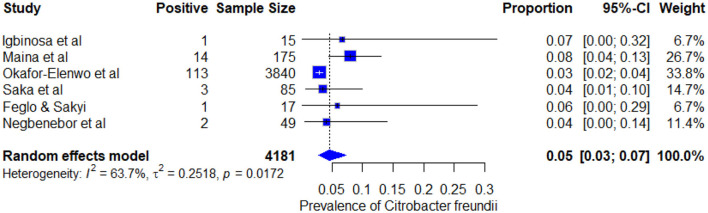
Pooled prevalence of *Citrobacter freundii* isolated from ready-to-eat (RTE) vegetable salads across Africa. CI, confidence interval.

**Figure 11:**
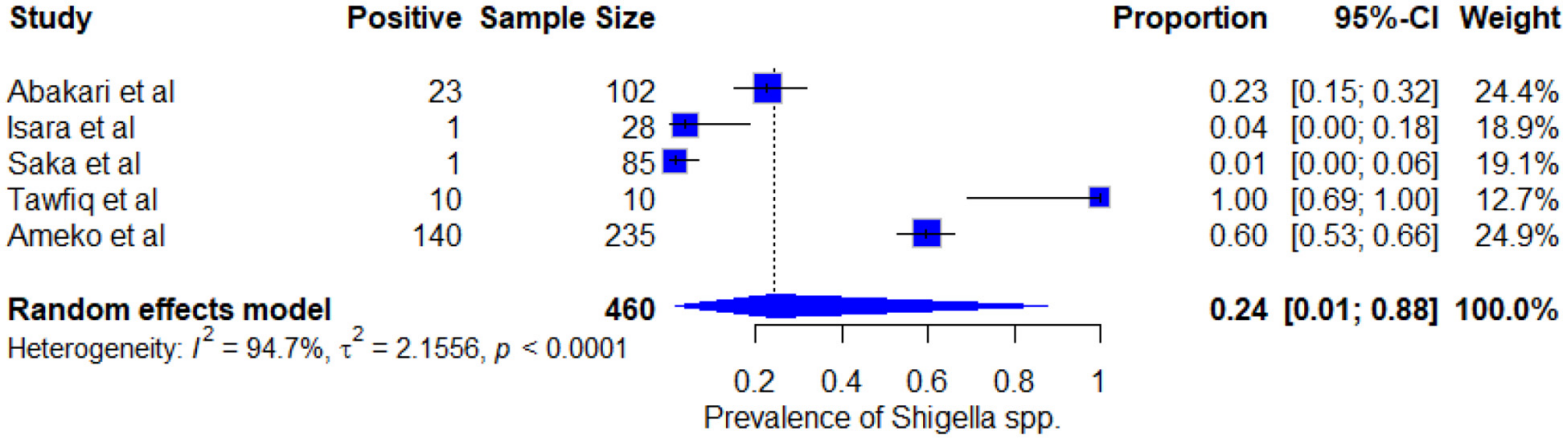
Pooled prevalence of *Shigella spp isolated* from ready-to-eat (RTE) vegetable salads across Africa. CI, confidence interval.

The meta-analytic pooled prevalences revealed variability in the frequency of bacterial contamination in ready-to-eat vegetable salads across Africa (Figures 3–11). *E. coli*, one of the most frequently reported organisms (17 studies), showed a pooled prevalence of 40% (95% CI: 20–64%; *I*^2^ = 84.5%), while *Salmonella* spp. (18 studies) had a pooled prevalence of 32% (95% CI: 16–54%; *I*^2^ = 92.3%). Among Gram-positive bacteria, *B. cereus* demonstrated a pooled prevalence of 28% (95% CI: 14–48%; *I*^2^ = 94.7%) across 9 studies, while *Staphylococcus* spp. (10 studies) had a pooled prevalence of 26% (95% CI: 13–47%; *I*^2^ = 97.0%). Other organisms such as *Klebsiella* spp., *Enterobacter* spp., *P. aeruginosa*, and *C. freundii* showed comparatively lower pooled prevalence estimates. Although *Shigella* spp. met the inclusion threshold (5 studies), its pooled estimate (24%; 95% CI: 1–88%) exhibited a very wide confidence interval, indicating substantial uncertainty; therefore, this estimate should be interpreted with caution. The statistical heterogeneity across the pooled estimates varied significantly by bacterial species. Based on the *I*^2^ statistic, heterogeneity was categorized as low for *P. aeruginosa* (28.9%), moderate for *K. oxytoca* (56.2%), *C. freundii* (63.7%), and *Enterobacter* spp. (67.0%), and high (*I*^2^ > 75%) for *Salmonella* spp. (92.3%), *E. coli* (84.5%), *Staphylococcus* spp. (97.0%), *B. cereus* (94.7%), and *Shigella* spp. (94.7%).

### Sensitivity analysis

Given the substantial heterogeneity observed in the majority of the analyses, a leave-one-out sensitivity analysis was conducted to explore whether specific studies were acting as outliers or disproportionately influencing the pooled prevalence and the *I*^2^ values (Tables 3–11). The analysis revealed that the pooled prevalence estimates for dominant isolates remained remarkably stable, with no single study exerting a disproportionate influence on the overall results. However, for *Klebsiella oxytoca*, the exclusion of Maina et al. reduced heterogeneity from 56.2% to 27.2%, while for *Pseudomonas aeruginosa*, the exclusion of Igbinosa et al. reduced *I*^2^ to 0%. Similarly, for Citrobacter freundii, removing Maina et al. or Okafor-Elenwo et al. eliminated statistical heterogeneity (*I*^2^ = 0%).

**Table 3:** Leave-one-out sensitivity analysis of *Salmonella* spp.

Study	Proportion	95%CI	*I^2^*
Gadaga et al.	0.326	0.172–0.528	92.67%
Christison et al.	0.299	0.164–0.481	92.58%
Abakari et al.	0.331	0.176–0.533	92.54%
Karikari et al.	0.291	0.16–0.468	91.57%
Igbinosa et al.	0.331	0.177–0.531	92.68%
Maina et al.	0.343	0.19–0.539	91.25%
Ajuzieogu et al.	0.333	0.179–0.534	92.61%
Isara et al.	0.325	0.173–0.527	92.71%
Mohammed and Muhammad	0.293	0.16–0.473	87.23%
Umar et al.	0.277	0.163–0.431	92.26%
Saka et al.	0.349	0.199–0.535	92%
Ladan et al.	0.337	0.182–0.536	92.54%
Ndunguru and Ndossi	0.34	0.189–0.533	92.60%
Ameko et al.	0.323	0.171–0.526	92.71%
Obajuluwa et al.	0.341	0.187–0.538	92.44%
Toe et al.	0.279	0.163–0.435	92.30%
Adeshina et al.	0.327	0.175–0.528	92.70%
Bukar et al.	0.335	0.18–0.535	92.60%

CI, confidence interval.

**Table 4:** Leave-one-out sensitivity analysis of *E. coli*.

Study	Proportion	95%CI	*I^2^*
Toe et al.	0.338	0.227–0.471	79.90%
Abakari et al.	0.411	0.226–0.625	85.36%
Karikari et al.	0.414	0.229–0.626	85.27%
Mbae et al.	0.361	0.212–0.543	82.78%
Igbinosa et al.	0.407	0.223–0.62	85.45%
Maina et al.	0.414	0.229–0.627	84.66%
Ajuzieogu et al.	0.418	0.235–0.628	85%
Saka et al.	0.412	0.227–0.625	85.31%
Ladan et al.	0.362	0.21–0.547	79.56%
Ndunguru and Ndossi	0.394	0.215–0.606	85%
Feglo and Sakyi	0.426	0.25–0.623	84.89%
Oyeyipo et al.	0.402	0.219–0.617	85.13%
Obajuluwa et al.	0.408	0.224–0.622	85.45%
Adeshina et al.	0.406	0.224–0.619	85.45%
Bukar et al.	0.405	0.222–0.62	85.42%
Negbenebor et al.	0.433	0.265–0.619	83.69%
Pesewu et al.	0.406	0.222–0.621	85.41%

CI, confidence interval.

**Table 5:** Leave-one-out sensitivity analysis of *Klebsiella oxytoca*.

Study	Proportion	95%Cl	*I^2^*
Igbinosa et al.	0.18	0.134–0.237	57.72%
Nyenje et al.	0.173	0.124–0.235	60.39%
Maina et al.	0.161	0.127–0.201	27.19%
Ajuzieogu et al.	0.17	0.121–0.232	60.55%
Saka et al.	0.183	0.133–0.245	54.20%
Ndunguru and Ndossi	0.173	0.126–0.233	60.57%
Feglo and Sakyi	0.165	0.122–0.22	56.09%
Oyeyipo et al.	0.176	0.126–0.239	59.24%
Obajuluwa et al.	0.184	0.137–0.243	55.07%
Negbenebor et al.	0.19	0.145–0.244	45.49%
Pesewu et al.	0.173	0.123–0.238	59.71%

**Table 6:** Leave-one-out sensitivity analysis of *Staphylococcus spp*.

Study	Proportion	95%CI	*I^2^*
Ajuzieogu et al.	0.291	0.151–0.486	97.32%
Isara et al.	0.245	0.119–0.44	97.04%
Okafor-Elenwo et al.	0.312	0.176–0.491	85.78%
Saka et al.	0.266	0.128–0.472	97.16%
Ndunguru and Ndossi	0.277	0.139–0.476	97.32%
Feglo and Sakyi	0.271	0.134–0.473	97.31%
Obajuluwa et al.	0.242	0.117–0.433	96.81%
Adeshina et al.	0.263	0.128–0.463	97.29%
Negbenebor et al.	0.21	0.121–0.34	95.82%
Pesewu et al.	0.253	0.121–0.455	96.85%

CI, confidence interval.

**Table 7:** Leave-one-out sensitivity analysis of *Bacillus cereus*.

Study	Proportion	95%CI	*I^2^*
Gadaga et al.	0.219	0.157–0.297	75.14%
Abakari et al.	0.273	0.137–0.471	95.41%
Isara et al.	0.282	0.145–0.476	95.39%
Mosupye et al.	0.249	0.138–0.407	95.34%
Okafor-Elenwo et al.	0.267	0.133–0.464	94.71%
Saka et al.	0.298	0.159–0.487	95.06%
Ndunguru and Ndossi	0.298	0.167–0.473	95.34%
Feglo and Sakyi	0.286	0.15–0.476	95.39%
Pesewu et al.	0.296	0.158–0.487	95.13%

CI, confidence interval.

**Table 8:** Leave-one-out sensitivity analysis of *Enterobacter spp*.

Study	Proportion	95%CI	*I^2^*
Igbinosa et al.	0.115	0.058–0.215	69.97%
Nyenje et al.	0.098	0.046–0.199	71.68%
Maina et al.	0.094	0.045–0.186	65.85%
Ajuzieogu et al.	0.114	0.055–0.222	67.72%
Saka et al.	0.145	0.088–0.228	52.41%
Feglo and Sakyi	0.117	0.06–0.216	69.48%
Oyeyipo et al.	0.094	0.047–0.179	68.23%
Negbenebor et al.	0.122	0.061–0.227	64.23%

CI, confidence interval.

**Table 9:** Leave-one-out sensitivity analysis of *Pseudomonas aeruginosa*.

Study	Proportion	95%CI	*I^2^*
Igbinosa et al.	0.101	0.075–0.135	0%
Isara et al.	0.122	0.083–0.175	28.74%
Saka et al.	0.115	0.07–0.185	39.16%
Ameko et al.	0.136	0.093–0.196	8.01%
Oyeyipo et al.	0.118	0.074–0.185	42.81%
Adeshina et al.	0.113	0.077–0.162	39.60%

CI, confidence interval.

**Table 10:** Leave-one-out sensitivity analysis of *Citrobacter freundii*.

Study	Proportion	95%CI	*I^2^*
Igbinosa et al.	0.044	0.026–0.075	69.82%
Maina et al.	0.03	0.025–0.036	0%
Okafor-Elenwo et al.	0.063	0.039–0.099	0%
Saka et al.	0.047	0.026–0.085	70.90%
Feglo and Sakyi	0.044	0.026–0.076	70.19%
Negbenebor et al.	0.046	0.026–0.081	70.74%

CI, confidence interval.

**Table 11:** Leave-one-out sensitivity analysis of *Shigella spp*.

Study	Proportion	95%CI	*I^2^*
Abakari et al.	0.244	0.013–0.892	92.22%
Isara et al.	0.344	0.03–0.9	94.94%
Saka et al.	0.4	0.062–0.871	94.06%
Tawfiq et al.	0.124	0.017–0.536	95.37%
Ameko et al.	0.17	0.01–0.806	86.64%

CI, confidence interval.

## Discussion

This systematic review and meta-analysis provide the most comprehensive synthesis to date of bacterial contamination in ready-to-eat (RTE) vegetable salads across Africa. By consolidating evidence from 31 studies spanning multiple African subregions, the analysis highlights both the diversity and public health relevance of bacterial communities, including both pathogenic and non-pathogenic organisms, contaminating salads.

The geographical distribution of included studies was heavily skewed toward West Africa, particularly Nigeria and Ghana, which together accounted for more than two-thirds of the available evidence. This concentration likely reflects higher research output, greater recognition of food safety challenges associated with informal food markets, and the dominance of street-vended RTE foods in these countries. However, this imbalance has important implications for interpretation. Pooled prevalence estimates may disproportionately represent contamination scenarios characteristic of densely populated West African urban markets, where regulatory oversight, cold-chain infrastructure, and access to potable water are often limited (Pesewu et al., 2014; Toe et al., 2017). In contrast, the relative scarcity of data from Central and North Africa constrains continent-wide inference and highlights major surveillance gaps. These findings represent a synthesis of available evidence and should not be interpreted as fully representative of the entire African continent due to geographic imbalance and high heterogeneity.

### Bacterial profile and contamination pathways

The predominance of Gram-negative bacteria observed in this synthesis is consistent with the known ecology of fresh produce and reflects multiple contamination routes along the farm-to-fork continuum (Kabir et al., 2023; Mesbah Zekar et al., 2017; Thomas et al., 2024). Enteric organisms such as *Escherichia coli, Salmonella* spp., and *Shigella* spp. are widely recognized indicators of fecal contamination and are commonly linked to pre-harvest factors, including the use of contaminated irrigation water, untreated organic fertilizers, and proximity to livestock. Studies from East and West Africa have repeatedly documented the use of wastewater or surface water for vegetable irrigation, particularly in peri-urban agriculture, creating conditions conducive to early contamination that may persist to the point of consumption (Ali et al., 2023; Darko et al., 2025).

It is important to distinguish between bacterial presence and public health risk in the context of ready-to-eat vegetable salads. Several organisms identified in this review, including Klebsiella spp., Enterobacter spp., Pseudomonas aeruginosa, Citrobacter freundii, and Proteus spp., are commonly part of the natural microbiota of plants and the surrounding environment (Kabir et al., 2023; Mesbah Zekar et al., 2017; Thomas et al., 2024). Their detection in raw vegetables is therefore not unexpected and does not necessarily indicate a direct risk of foodborne disease. These organisms are primarily opportunistic pathogens and are rarely implicated in classical foodborne outbreaks.

In contrast, the frequent detection of Gram-positive organisms such as *Staphylococcus* spp. and *Bacillus cereus* suggests a strong contribution from post-harvest contamination. *Staphylococcus* species are closely associated with human skin, nasal carriage, and food handlers, implicating poor personal hygiene and cross-contamination during washing, chopping, and mixing of salads (Viana et al., 2025; Akinnola et al., 2022). *B. cereus*, a spore-forming organism ubiquitous in soil and dust, may be introduced during harvesting or processing and subsequently proliferate under ambient storage conditions (Jessberger et al., 2020). Although Bacillus cereus was frequently reported, its public health significance depends on bacterial load rather than mere presence. Foodborne illness associated with B. cereus typically requires concentrations exceeding 105 CFU/g, levels that are not commonly reported in fresh ready-to-eat vegetables. In this context, its detection may primarily reflect environmental contamination from soil rather than an immediate risk of intoxication. Furthermore, outbreaks linked to fresh vegetable salads are rarely reported. The co-occurrence of enteric and handling-associated bacteria within the same food matrix highlights the cumulative risk posed by multiple, overlapping contamination points.

The pooled prevalence estimates revealed substantial variability across bacterial species, with consistently high heterogeneity for most outcomes. This heterogeneity likely reflects real differences in contamination intensity rather than methodological artifact alone. Variations in study design, sample size, detection methods, and reporting formats undoubtedly contributed to statistical heterogeneity; however, contextual factors appear equally important. Studies conducted among street-vended or market-sold salads generally reported higher prevalence than those from restaurants or institutional catering, mirroring findings from other low- and middle-income countries where informal food vending dominates (Muyanja et al., 2011; Nortey et al., 2024; Desye et al., 2023). Differences in washing practices, reuse of rinse water, absence of refrigeration, and prolonged holding times under warm conditions further amplify bacterial growth and survival.

The detection of *L. monocytogenes* across different settings remains epidemiologically significant and compares with studies from other countries, which report sporadic but consequential contamination of RTE vegetables with Listeria, particularly in environments lacking cold-chain control and effective sanitation (Niu et al., 2024; Chuks and Anayo, 2012). When contrasted with high-income countries, where prevalence in RTE salads is typically low but stringently monitored, the African context reflects a higher potential for unnoticed exposure due to limited routine surveillance (Dufailu et al., 2021; Williams et al., 2025).

Moderate pooled prevalence estimates for *E. coli* and *Salmonella* spp. are broadly consistent with findings from other African and LMIC settings, reinforcing their role as sentinel organisms for fecal contamination in fresh produce. These pathogens have also been implicated in numerous produce-associated outbreaks globally, emphasizing that salads represent a well-established vehicle for foodborne transmission when control measures fail (Kuboka et al., 2026; Ali et al., 2023; Olaitan et al., 2025; Moses et al., 2016).

### Public health relevance and risk implications

From a public health perspective, our findings highlight the presence of a mixture of organisms, including established foodborne pathogens as well as environmental and opportunistic bacteria. The public health risk associated with these organisms is context-dependent and influenced by factors such as bacterial load, virulence characteristics, and host susceptibility.

### Implications for food safety policy and surveillance

The findings of this study highlight weaknesses in food safety systems governing RTE vegetable salads in Africa. Current regulatory frameworks often focus on animal-source foods, with comparatively limited oversight of fresh produce and informal food vending. Strengthening surveillance for microbial contamination of RTE salads, particularly in high-risk urban markets, should be prioritized. Interventions must address both pre-harvest and post-harvest contamination pathways. At the production level, improved management of irrigation water quality and safe use of organic fertilizers are essential. At the retail and preparation stages, targeted hygiene training for food handlers (World Health Organization and Food and Agriculture Organization of the United Nations. 2023), access to potable water, and basic infrastructure improvements could substantially reduce contamination.

### Strengths and limitations

The strengths of this review lie in its comprehensive search strategy, adherence to PRISMA guidelines, and application of meta-analytic techniques to quantify bacterial prevalence across a diverse set of African studies. Nevertheless, limitations must be acknowledged. The uneven geographic distribution of studies and high heterogeneity constrain definitive inference regarding continent-wide patterns. Despite these limitations, the synthesis provides a critical evidence base for understanding contamination patterns and identifying priority areas for intervention.

### Conclusions

This study demonstrates the presence of diverse bacterial communities in ready-to-eat vegetable salads across Africa, including both established foodborne pathogens and organisms commonly associated with plant microbiota. While the detection of certain pathogens raises food safety concerns, the presence of environmental and opportunistic bacteria reflects contributions from both pre-harvest sources, such as irrigation water and soil, and post-harvest practices, including food handling and storage. Although prevalence estimates varied widely across studies, the overall evidence suggests that ready-to-eat salads may serve as a potential exposure pathway for foodborne infections. These findings represent a synthesis of available evidence and should not be interpreted as fully representative of the entire African continent due to geographic imbalance and high heterogeneity. Strengthening microbiological surveillance, hygiene practices, and regulatory oversight for fresh produce is essential to reduce consumer risk.

## Data Availability

The original contributions presented in the study are included in the article/Supplementary material, further inquiries can be directed to the corresponding author.
