# Supplementary material for: A systematic review and meta-analysis of the bacterial profile of ready-to-eat vegetable salads across Africa
Source: Front Microbiol. 2026 Jun 3;17:1802185. doi: 10.3389/fmicb.2026.1802185 (PMC13272457; doi:10.3389/fmicb.2026.1802185)
Supplement: Supplementary file 1 [file Table_1.DOCX]

| Database | Search Query | Search Date |
| --- | --- | --- |
| PubMed | (("ready to eat"[Title/Abstract] OR "ready-to-eat"[Title/Abstract] OR "minimally processed"[Title/Abstract] OR "fresh produce"[Title/Abstract] OR "raw vegetable*"[Title/Abstract] OR salad*[Title/Abstract] OR lettuce[Title/Abstract] OR cabbage[Title/Abstract] OR carrot*[Title/Abstract] OR tomato*[Title/Abstract] OR vegetable*[Title/Abstract]) AND (bacter*[Title/Abstract] OR microb*[Title/Abstract] OR "foodborne pathogen*"[Title/Abstract] OR "Escherichia coli"[Title/Abstract] OR Salmonella[Title/Abstract] OR Listeria[Title/Abstract] OR Staphylococcus[Title/Abstract] OR "food contamination"[Title/Abstract] OR "food microbiology"[Title/Abstract]) AND (Algeria[Title/Abstract] OR Angola[Title/Abstract] OR Benin[Title/Abstract] OR Botswana[Title/Abstract] OR "Burkina Faso"[Title/Abstract] OR Burundi[Title/Abstract] OR Cameroon[Title/Abstract] OR "Cape Verde"[Title/Abstract] OR "Central African Republic"[Title/Abstract] OR Chad[Title/Abstract] OR Comoros[Title/Abstract] OR Congo[Title/Abstract] OR "Democratic Republic of the Congo"[Title/Abstract] OR Djibouti[Title/Abstract] OR Egypt[Title/Abstract] OR "Equatorial Guinea"[Title/Abstract] OR Eritrea[Title/Abstract] OR Eswatini[Title/Abstract] OR Ethiopia[Title/Abstract] OR Gabon[Title/Abstract] OR Gambia[Title/Abstract] OR Ghana[Title/Abstract] OR Guinea[Title/Abstract] OR "Guinea-Bissau"[Title/Abstract] OR "Côte d'Ivoire"[Title/Abstract] OR Kenya[Title/Abstract] OR Lesotho[Title/Abstract] OR Liberia[Title/Abstract] OR Libya[Title/Abstract] OR Madagascar[Title/Abstract] OR Malawi[Title/Abstract] OR Mali[Title/Abstract] OR Mauritania[Title/Abstract] OR Mauritius[Title/Abstract] OR Morocco[Title/Abstract] OR Mozambique[Title/Abstract] OR Namibia[Title/Abstract] OR Niger[Title/Abstract] OR Nigeria[Title/Abstract] OR Rwanda[Title/Abstract] OR Senegal[Title/Abstract] OR Seychelles[Title/Abstract] OR "Sierra Leone"[Title/Abstract] OR Somalia[Title/Abstract] OR "South Africa"[Title/Abstract] OR Sudan[Title/Abstract] OR "South Sudan"[Title/Abstract] OR Tanzania[Title/Abstract] OR Togo[Title/Abstract] OR Tunisia[Title/Abstract] OR Uganda[Title/Abstract] OR Zambia[Title/Abstract] OR Zimbabwe[Title/Abstract])) |  |
| Scopus | TITLE-ABS-KEY(("ready to eat" OR "ready-to-eat" OR "minimally processed" OR "fresh produce" OR "raw vegetable*" OR salad* OR lettuce OR cabbage OR carrot* OR tomato* OR vegetable*) AND (bacter* OR microb* OR "foodborne pathogen*" OR "Escherichia coli" OR Salmonella OR Listeria OR Staphylococcus OR "food contamination" OR "food microbiology") AND (Algeria OR Angola OR Benin OR Botswana OR "Burkina Faso" OR Burundi OR Cameroon OR "Cape Verde" OR "Central African Republic" OR Chad OR Comoros OR Congo OR "Democratic Republic of the Congo" OR Djibouti OR Egypt OR "Equatorial Guinea" OR Eritrea OR Eswatini OR Ethiopia OR Gabon OR Gambia OR Ghana OR Guinea OR "Guinea-Bissau" OR "Côte d'Ivoire" OR Kenya OR Lesotho OR Liberia OR Libya OR Madagascar OR Malawi OR Mali OR Mauritania OR Mauritius OR Morocco OR Mozambique OR Namibia OR Niger OR Nigeria OR Rwanda OR Senegal OR Seychelles OR "Sierra Leone" OR Somalia OR "South Africa" OR Sudan OR "South Sudan" OR Tanzania OR Togo OR Tunisia OR Uganda OR Zambia OR Zimbabwe)) |  |
| Google Scholar | ("ready to eat" OR "ready-to-eat" OR "minimally processed" OR "fresh produce" OR "raw vegetables" OR salads OR lettuce OR cabbage OR carrots OR tomatoes OR vegetables) AND (bacteria OR microbial OR microbiology OR "foodborne pathogens" OR "Escherichia coli" OR Salmonella OR Listeria OR Staphylococcus OR "food contamination") AND (Algeria OR Angola OR Benin OR Botswana OR "Burkina Faso" OR Burundi OR Cameroon OR "Cape Verde" OR "Central African Republic" OR Chad OR Comoros OR Congo OR "Democratic Republic of the Congo" OR Djibouti OR Egypt OR "Equatorial Guinea" OR Eritrea OR Eswatini OR Ethiopia OR Gabon OR Gambia OR Ghana OR Guinea OR "Guinea-Bissau" OR "Cote d'Ivoire" OR Kenya OR Lesotho OR Liberia OR Libya OR Madagascar OR Malawi OR Mali OR Mauritania OR Mauritius OR Morocco OR Mozambique OR Namibia OR Niger OR Nigeria OR Rwanda OR Senegal OR Seychelles OR "Sierra Leone" OR Somalia OR "South Africa" OR Sudan OR "South Sudan" OR Tanzania OR Togo OR Tunisia OR Uganda OR Zambia OR Zimbabwe) |  |
|  |  |  |
